# Development and external validation of a LASSO-based parsimonious nomogram for predicting BPPV recurrence: a multi-center retrospective cohort study

**DOI:** 10.3389/fneur.2026.1812718

**Published:** 2026-06-15

**Authors:** Qiaozhi Jin, Ziyuan Chen, Yong Li, Yilong Wang, Changyu Duan, Yongjie Ying, Fude Jin

**Affiliations:** 1 Department of Otolaryngology, Taizhou Municipal Hospital (Taizhou University Affiliated Municipal Hospital), Taizhou, China; 2 School of Medicine, Taizhou University, Taizhou, China; 3 Department of Otorhinolaryngology-Head and Neck Surgery, The First Affiliated Hospital of Fujian Medical University, Fuzhou, China; 4 Department of Otolaryngology, The People’s Hospital of Yuhuan, Taizhou, China

**Keywords:** Benign Paroxysmal Positional Vertigo, external validation, LASSO regression, nomogram, parsimonious model, recurrence

## Abstract

**Background:**

Benign Paroxysmal Positional Vertigo (BPPV) exhibits high recurrence rates post-repositioning. This study aimed to identify core risk factors and develop a parsimonious, externally validated nomogram to personalize recurrence risk stratification.

**Methods:**

We conducted a multi-center retrospective cohort study involving BPPV patients from two medical centers (2020–2025). The Least Absolute Shrinkage and Selection Operator (LASSO) and stepwise multivariate logistic regression were employed to identify predictors and construct the model. Performance was evaluated using the Area under the Curve (AUC), calibration plots, Decision Curve Analysis (DCA), and Clinical Impact Curves (CIC).

**Results:**

Among the participants, the 1-year recurrence rate was 23.97%. A parsimonious model comprising three core variables: Diabetes Mellitus (OR = 11.42), Non-posterior canal involvement (OR = 4.17), and 25-hydroxyvitamin D deficiency [25 (OH) D] (OR = 3.76), was established. The model demonstrated good discrimination, with an AUC of 0.905 in the training set, 0.872 in the internal validation set, and 0.853 in the external validation set. Calibration curves showed excellent agreement between predicted and observed probabilities. DCA and CIC confirmed significant net clinical benefit across a wide range of threshold probabilities (0.05–0.76), outperforming default clinical strategies.

**Conclusion:**

We developed and externally validated a highly accurate nomogram integrating metabolic, anatomical, and nutritional markers. This simplified tool facilitates early identification of high-risk patients and supports targeted secondary prevention in clinical practice.

## 1 Introduction

Benign Paroxysmal Positional Vertigo (BPPV) represents the most prevalent peripheral vestibular disorder globally, accounting for approximately 17%–42% of diagnoses in specialized vestibular clinics and an estimated lifetime prevalence of 2.4% (1–3). The established pathophysiology involves the dislodgment of otoconia from the utricular macula, which subsequently migrate into the semicircular canals (canalithiasis) or adhere to the cupula (cupulolithiasis) under gravitational forces. This displacement precipitates intense, paroxysmal vertigo and characteristic nystagmus elicited by changes in head position (4). While Canalith Repositioning Procedures (CRP), such as the Epley and Barbecue maneuvers, serve as the primary clinical intervention with immediate remission rates exceeding 90%, the high incidence of recurrence constitutes a substantial challenge in longitudinal management (5, 6). Current literature suggests that the 1-year recurrence rate following successful CRP ranges from 15 to 30%, with the 5-year cumulative incidence approaching 50% (7, 8). The unpredictable and repetitive nature of these recurrences imposes a dual burden: it not only elevates the risk of physical sequelae, such as falls and fractures in the elderly, but also frequently exacerbates a cycle of “vestibular-psychological distress,” characterized by persistent anxiety and residual dizziness (9–11). Consequently, precise risk stratification is of paramount clinical importance for formulating individualized secondary prevention strategies.

Despite a growing body of research investigating predictors of BPPV recurrence, findings remain considerably heterogeneous. Previous studies have implicated systemic factors, including advanced age, female gender, osteoporosis, and metabolic comorbidities (e.g., hypertension, diabetes mellitus, and hyperlipidemia), as potential contributors to otoconial detachment (12–14). Notably, the association between 25-hydroxyvitamin D [25(OH)D] deficiency and otoconial metabolic dysregulation has become a focal point of investigation, with multiple meta-analyses identifying reduced serum 25(OH)D levels as an independent predictor of recurrence (15–17). Beyond systemic metabolic factors, local anatomical variations, such as the specific semicircular canal affected, and neuropsychological status have also garnered increasing scrutiny (18, 19). However, extant literature has predominantly focused on these risk factors in isolation, often failing to account for the complex interplay among metabolic disturbances, anatomical variations, and psychological determinants. There remains a paucity of comprehensive prognostic assessment systems capable of integrating these multifaceted characteristics to holistically evaluate recurrence risk.

Nomograms, which serve as graphical representations of statistical predictive models, facilitate the translation of complex regression equations into intuitive scoring systems. While these tools have been widely implemented in oncology for prognostic prediction (20–22), their application in BPPV remains limited. The few existing nomograms addressing BPPV recurrence are constrained by distinct limitations: most rely heavily on basic demographic indicators while insufficiently accounting for specific clinical subtypes. Critically, these models often lack rigorous multi-center external validation, rendering their generalizability and clinical applicability across diverse medical settings uncertain (23, 24).

To address these critical knowledge gaps, we conducted a multi-center retrospective cohort study utilizing large-scale clinical data from Taizhou Municipal Hospital and Yuhuan People’s Hospital. The objective was to systematically elucidate the key prognostic determinants of recurrence following successful CRP. We employed the Least Absolute Shrinkage and Selection Operator (LASSO) regression for dimensionality reduction to mitigate the effects of multicollinearity, subsequently utilizing multivariate logistic regression to construct the predictive model. Distinct from prior studies, we prioritized the development and validation of a “parsimonious model” based on core indicators. By rigorously evaluating the discrimination, calibration, and net clinical benefit of the model within both internal and external independent validation cohorts, we aim to provide clinicians with a risk screening tool that balances predictive accuracy with clinical feasibility, thereby offering a framework for precision follow-up and stratified intervention.

## 2 Materials and methods

### 2.1 Study population

We conducted a multi-center retrospective study involving patients diagnosed with BPPV at the Departments of Otolaryngology of Taizhou Municipal Hospital and The People’s Hospital of Yuhuan between January 2020 and December 2025. A total of 680 patients from Taizhou Municipal Hospital were randomly partitioned into a Training Set and an Internal Validation Set at a ratio of 7:3 using a random number table. Additionally, an independent cohort of 100 patients from Yuhuan People’s Hospital was selected as the External Validation Set to evaluate the model’s generalizability.

#### 2.1.1 Inclusion criteria

**Figure 1:**
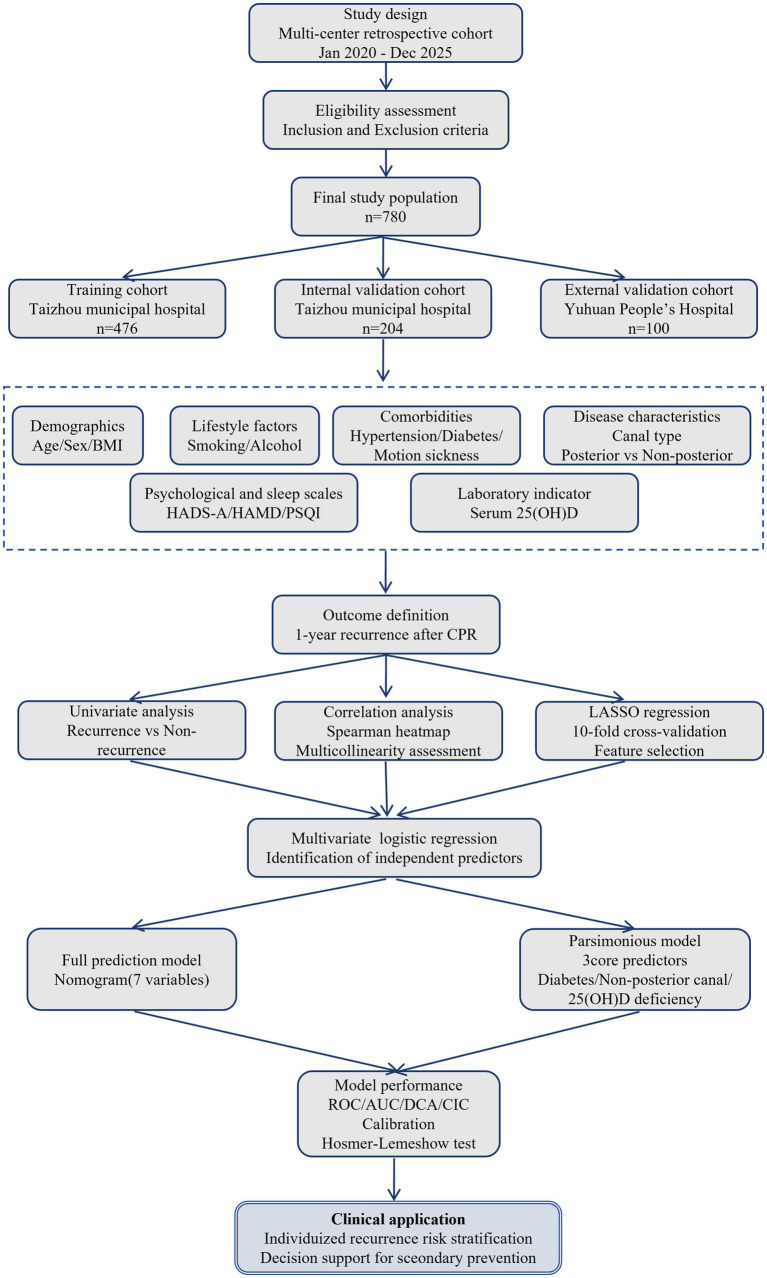
Study workflow and model development strategy for predicting BPPV recurrence.

(i) A confirmed diagnosis of BPPV based on the Guideline for Diagnosis and Treatment of Benign Paroxysmal Positional Vertigo (2017 Edition, China) (25), verified by a positive Dix-Hallpike test and/or Roll test, (ii) Receipt of at least one standardized CRP, (iii) Availability of complete clinical data, including demographic characteristics, comorbidities, scale assessments, and serum 25(OH)D levels, (iv) A follow-up duration of 12 months. Research workflow is shown in Figure 1.

#### 2.1.2 Exclusion criteria

(i) Co-existence of other vestibular disorders (e.g., Meniere’s disease, vestibular neuritis), cervical spondylosis, or cerebrovascular diseases, (ii) Severe dysfunction of vital organs (e.g., heart, liver, or kidney failure), (iii) A history of severe psychiatric disorders, such as schizophrenia or bipolar disorder, (iv) Loss to follow-up or death due to other causes during the study period. This study was approved by the Ethics Committees of Taizhou Municipal Hospital (Approval No. LWYJ2026323) and Yuhuan People’s Hospital (Approval No. 2026007). The requirement for informed consent was waived due to the retrospective nature of the study.

### 2.2 Data collection

Clinical data were systematically extracted from the electronic medical record (EMR) and laboratory information systems (LIS). The collected variables included: (1) Demographic Characteristics: Gender, age, and Body Mass Index (BMI), (2) Comorbidities: Hypertension (defined as a confirmed history or systolic blood pressure≥140 mmHg and/or diastolic blood pressure≥90 mmHg); Diabetes Mellitus (defined as a confirmed history, fasting blood glucose≥7.0 mmol/L, or 2-h postprandial blood glucose≥11.1 mmol/L); and Motion Sickness (defined as a history of motion intolerance characterized by autonomic symptoms in response to vehicular travel or visual motion stimuli), (3) Disease-related Characteristics: Type of affected semicircular canal, which was dichotomized into posterior canal and non-posterior canal BPPV for primary analysis. This classification is clinically justified as posterior canal BPPV accounts for 80%–90% of all cases; non-posterior canal BPPV included horizontal, anterior, multicanal and bilateral BPPV, and further subdivision was not performed due to small numbers of individual subtypes, which would lead to insufficient statistical power for meaningful subgroup comparisons, (4) Scale Assessments: Anxiety was evaluated using the Hospital Anxiety and Depression Scale-Anxiety subscale (HADS-A; scores≥8 indicate suspected or definite anxiety). Depression was assessed using the Hamilton Depression Rating Scale (HAMD; scores≥7 indicate depression). Sleep quality was measured using the Pittsburgh Sleep Quality Index (PSQI; scores≥5 indicate poor sleep quality), (5) Laboratory Indicators: Serum 25(OH)D levels (deficiency defined as < 20 ng/mL),(6) Lifestyle Factors: History of smoking (cumulative smoking for≥1 year) and alcohol consumption (cumulative drinking for ≥1 year).

All patients received standardized canalith repositioning maneuvers tailored to the affected semicircular canal type. Successful CRP was defined as complete resolution of positional vertigo and disappearance of positional nystagmus on follow-up positional testing conducted 24–48 h after the final maneuver.

### 2.3 Follow-up and outcome definitions

All patients were followed up for 12 months commencing from the date of the initial successful CRP, using a standardized follow-up protocol uniformly implemented at both study centers. Scheduled in-person follow-up visits were arranged at 1 month, 3 months, 6 months, and 12 months after treatment. For patients who failed to attend scheduled in-person visits, telephone interviews were conducted as a screening tool to identify potential recurrence events. All patients reporting recurrent positional vertigo symptoms during either in-person visits or telephone interviews were required to return to the hospital for confirmatory positional testing (Dix-Hallpike test and/or supine roll test) within 72 h of symptom onset. Recurrence was defined as the re-emergence of positional vertigo (lasting seconds to tens of seconds) accompanied by nystagmus and confirmed by a positive positional test, following a period of complete symptom remission lasting ≥7 days. Based on the 1-year follow-up outcomes, patients were categorized into a Recurrence Group and a Non-recurrence Group.

### 2.4 Statistical analysis

Statistical analyses were performed using SPSS software (Version 26.0, IBM Corp, Armonk, NY) and R software (Version 4.2.1, The R Foundation). Continuous variables conforming to a normal distribution were expressed as mean ± standard deviation (*x* ± *s*) and compared between groups using the Student’s *t*-test. Non-normally distributed continuous variables were presented as median (interquartile range) [M(Q1, Q3)] and compared using the Mann–Whitney *U* test. Categorical variables were expressed as frequencies and percentages [*n*(%)] and analyzed using the Chi-square (*χ*^2^) test or Fisher’s exact test.

*Variable selection and model optimization*: variable selection was performed via a multi-step standardized pipeline compliant with the TRIPOD reporting guidelines. First, univariate logistic regression screening was conducted to exclude variables with poor predictive performance and negligible correlation with BPPV recurrence. This step reduced data dimensionality, decreased potential statistical noise, and improved the computational stability of the subsequent LASSO regression. Then, the least absolute shrinkage and selection operator (LASSO) regression with 10-fold cross-validation was applied to screen candidate variables. The optimal penalty parameter (*λ*) was determined at the minimum mean squared error. Utilizing the penalty shrinkage mechanism, the LASSO automatically suppressed redundant variables and mitigated the risk of overfitting by shrinking the coefficients of less important predictors toward zero. Finally, backward stepwise multivariate logistic regression was performed on the LASSO-selected variables to further optimize the model. This step retained only clinically meaningful and statistically independent predictors, enabling the establishment of a parsimonious prediction model suitable for practical clinical deployment. To reduce model optimism and instability potentially caused by the multi-step variable screening process, the LASSO shrinkage procedure was integral to the entire variable selection workflow. Moreover, strict internal cross-validation (via 10-fold cross-validation during LASSO tuning) and independent external validation were implemented to calibrate model performance and verify generalizability. The dual-cohort validation design served to further correct potential overfitting bias induced by the multi-step modeling process.

*Nomogram construction*: an individualized prognostic nomogram was established based on the identified core independent risk factors using the rms package in R.

*Model validation and evaluation*: (i) Discrimination: Evaluated by plotting Receiver Operating Characteristic (ROC) curves and calculating the AUC with 95% confidence intervals derived from 1,000 bootstrap resamples. (ii) Calibration: Assessed by plotting calibration curves and performing the Hosmer-Lemeshow goodness-of-fit test. (iii) Clinical Utility: Evaluated using DCA to calculate the net benefit at various threshold probabilities. Additionally, Clinical Impact Curves (CIC) were plotted to assess the concordance between the predicted high-risk population and the actual occurrence of events.

## 3 Results

### 3.1 Baseline characteristics of patients

A total of 680 eligible patients from Taizhou Municipal Hospital were enrolled and randomly assigned to a training cohort (*n* = 476) and an internal validation cohort (*n* = 204) using a 7:3 ratio. Furthermore, an independent cohort of 100 patients was recruited from Yuhuan People’s Hospital to serve as an external validation set. No statistically significant differences in baseline demographic or clinical characteristics were observed across the three cohorts (all *p* > 0.05), supporting inter-group comparability. During the 1-year follow-up period, 187 instances of recurrence were identified within the total study population (*N* = 780), representing an overall recurrence rate of 23.97%. Comparative analysis demonstrated that recurrence rates did not differ significantly among the training, internal validation, and external validation cohorts (all *p* > 0.05) (Table 1).

**Table 1:** Comparison of baseline characteristics between the training and validation sets.

Variable	Category	Training set (*n* = 476)	Internal validation set (*n* = 204)	External validation set (*n* = 100)	Test statistic (*x*^2^/*t*/*F*)	*p*-value
Gender					1.520	0.468
	Male	265 (55.67%)	89 (43.63%)	37 (37.00%)		
	Female	211 (44.33%)	115(56.37%)	63(63.00%)		
Age (years)		55.21 ± 11.36	53.58 ± 8.95	54.08 ± 10.77	0.579	0.561
BMI					1.614	0.806
	Normal	286 (60.08%)	130 (63.73%)	61 (61.00%)		
	Overweight	74 (15.55%)	33 (16.18%)	17 (17.00%)		
	Underweight	116 (24.37%)	41(20.10%)	22(22.00%)		
Smoking					0.171	0.918
	Yes	99 (20.80%)	45 (22.06%)	22 (22.00%)		
	No	377 (79.20%)	159 (77.94%)	78 (78.00%)		
Alcohol					0.408	0.816
	Yes	137 (28.78%)	63 (30.88%)	31 (31.00%)		
	No	339 (71.22%)	141 (69.12%)	69 (69.00%)		
Hypertension					0.123	0.940
	Yes	134 (28.15%)	60 (29.41%)	28 (28.00%)		
	No	342 (71.85%)	144 (70.59%)	72 (72.00%)		
Diabetes mellitus					0.209	0.901
	Yes	102 (21.43%)	41 (20.10%)	20 (20.00%)		
	No	374 (78.57%)	163 (79.90%)	80 (80.00%)		
Motion sickness					0.747	0.688
	Yes	96 (17.02%)	37 (18.14%)	17 (17.00%)		
	No	380 (82.98%)	167 (81.86%)	83 (83.00%)		
Non-posterior canal					0.534	0.766
	Yes	117 (24.58%)	45 (22.06%)	23 (23.00%)		
	No	359 (75.42%)	159 (77.94%)	77 (77.00%)		
HADS-A					3.436	0.179
	Elevated	128 (26.89%)	47 (23.04%)	33 (33.00%)		
	Normal	348 (73.11%)	157 (76.96%)	67 (67.00%)		
HAMD					2.855	0.240
	Elevated	108 (22.69%)	56 (27.45%)	29 (29.00%)		
	Normal	368 (77.31%)	148 (72.55%)	71 (71.00%)		
PSQI					3.406	0.182
	Elevated	139 (29.20%)	51 (25.00%)	21 (21.00%)		
	Normal	337 (70.80%)	153 (75.00%)	79 (79.00%)		
25(OH)D					4.508	0.105
	Low	133 (27.94%)	69 (33.82%)	37 (37.00%)		
	Normal	343 (72.06%)	135 (66.18%)	63 (63.00%)		
Recurrence					0.581	0.748
	Yes	117 (24.58%)	49 (24.02%)	21 (21.00%)		
	No	359 (75.42%)	155 (75.98%)	79 (79.00%)		

### 3.2 Univariate analysis of risk factors for recurrence

The results of the univariate analysis revealed significant differences in several clinical characteristics between the recurrence and non-recurrence groups. In the recurrence group, the proportions of hypertension, diabetes mellitus, motion sickness, and non-posterior semicircular canal BPPV were significantly higher than those in the non-recurrence group (all *p* < 0.05). Regarding clinical scale assessments, the HADS-A, HAMD, and PSQI scores were significantly higher in the recurrence group compared to the non-recurrence group. Furthermore, the incidence of 25(OH)D deficiency was significantly increased in the recurrence group (all *p* < 0.05). However, no statistically significant differences were observed between the two groups in terms of demographic characteristics or lifestyle habits, including gender, age, BMI, smoking history, and alcohol consumption (all *p* > 0.05), indicating a balanced distribution of these variables (Table 2).

**Table 2:** Univariate analysis of risk factors for BPPV recurrence in the training set.

Variable	Category	Recurrence group (*n* = 117)	Non-recurrence group (*n* = 359)	Test statistic (*χ*^2^/*t*)	*p*-value
Gender				3.835	0.0502
	Male	60 (51.28%)	147 (40.95%)		
	Female	57 (48.72%)	212 (59.05%)		
Age (years)		55.67 ± 9.92	55.06 ± 11.80	2.02	0.181
BMI				0.77	0.679
	Normal	67 (57.26%)	219 (61.00%)		
	Overweight	18 (15.38%)	56 (15.60%)		
	Underweight	32 (27.35%)	84 (23.40%)		
Smoking				0.92	0.336
	Yes	28 (23.93%)	71 (19.78%)		
	No	89 (76.07%)	288 (80.22%)		
Alcohol				2.97	0.085
	Yes	41 (35.04%)	96 (26.74%)		
	No	76 (64.96%)	263 (73.26%)		
Hypertension				81.17	<0.001
	Yes	71(60.68%)	63 (17.55%)		
	No	46(39.32%)	296 (82.45%)		
Diabetes mellitus				102.00	<0.001
	Yes	64 (54.70%)	38 (10.58%)		
	No	53 (45.30%)	321 (89.42%)		
Motion sickness				49.07	<0.001
	Yes	50 (42.74%)	46 (12.81%)		
	No	67 (57.26%)	313 (87.19%)		
Non-posterior canal				98.99	<0.001
	Yes	69 (58.97%)	48 (13.37%)		
	No	48 (41.03%)	311 (86.63%)		
HADS-A				53.76	<0.001
	Elevated	62 (52.99%)	66 (18.38%)		
	Normal	55 (47.01%)	293 (81.62%)		
HAMD				81.21	<0.001
	Elevated	62 (52.99%)	46 (12.81%)		
	Normal	55 (47.01%)	313 (87.19%)		
PSQI				74.37	<0.001
	Elevated	71 (60.68%)	68 (18.94%)		
	Normal	46 (39.32%)	291 (81.06%)		
25(OH)D				110.50	<0.001
	Low	77 (65.81%)	56 (15.60%)		
	Normal	40 (34.19%)	303 (84.40%)		

### 3.3 Multicollinearity diagnostics and correlation analysis of potential risk factors

**Figure 2:**
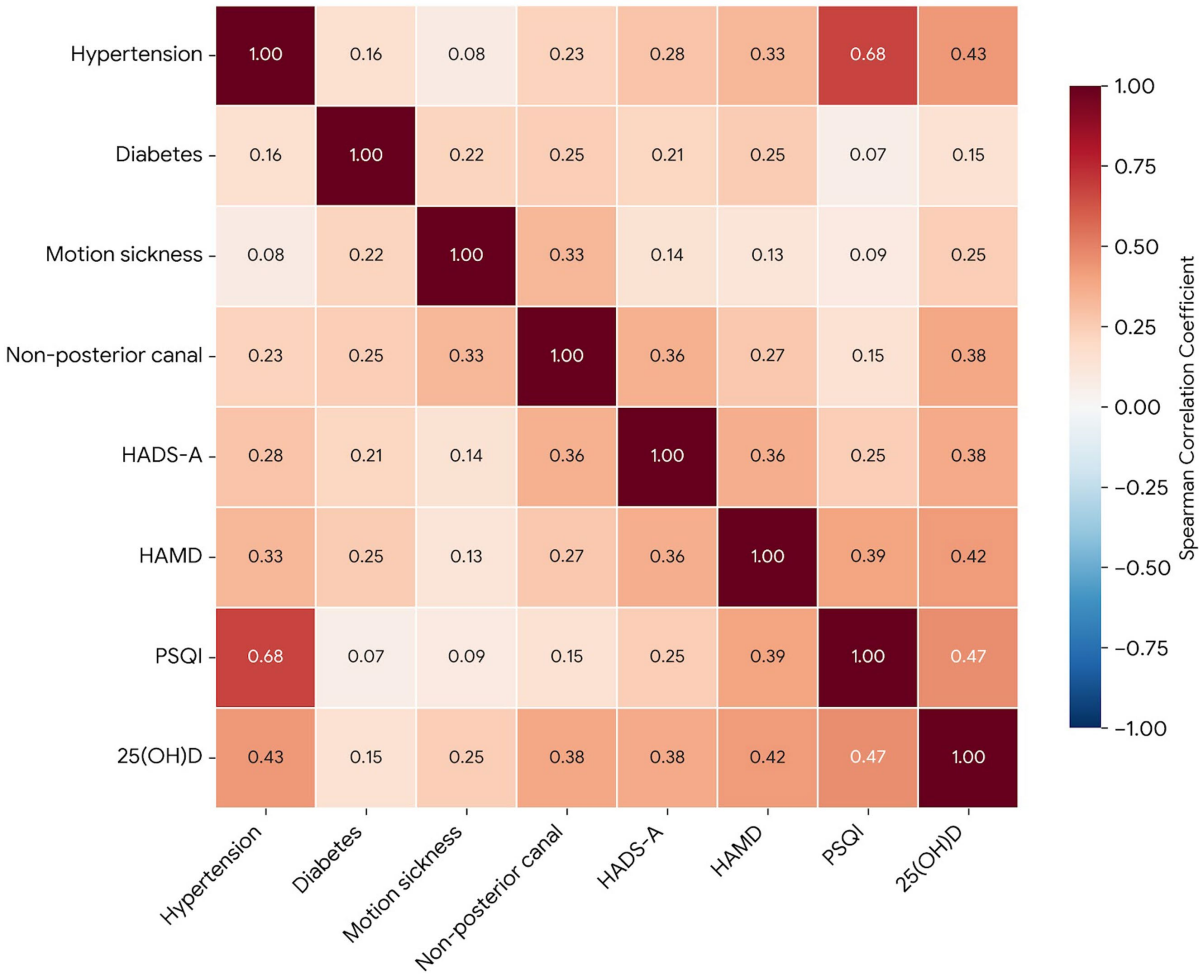
Heatmap of Spearman correlation analysis for potential risk factors of BPPV recurrence. The numbers within the grid represent the Spearman rank correlation coefficients (*r*) between pairs of variables. The color bar indicates the strength and direction of the correlations, with deeper colors representing stronger correlations.

To eliminate the potential interference of multicollinearity within the regression model and to elucidate the internal associations among various risk factors, Spearman rank correlation coefficients were employed to analyze the candidate predictors. The correlation matrix heatmap (Figure 2) demonstrated that although statistically significant linear relationships existed between certain variables, all pairwise correlation coefficients (*r*) remained below 0.70. This indicates an absence of substantial multicollinearity among the predictors, thereby satisfying the independence assumption requisite for multivariate logistic regression analysis. Further analysis revealed a significant positive correlation between hypertension and PSQI scores (*r* = 0.68), suggesting that diminished sleep quality is frequently concomitant with elevated blood pressure in patients with recurrent BPPV. Serum 25(OH)D levels exhibited broad interactive characteristics; specifically, 25(OH)D deficiency demonstrated moderate correlations with PSQI scores (*r* = 0.47), hypertension (*r* = 0.43), and HAMD scores (*r* = 0.42). These findings suggest that 25(OH)D insufficiency may serve as a common pathophysiological substrate for metabolic and neuropsychiatric manifestations. Additionally, a moderate positive correlation was observed between HADS-A and HAMD scores (*r* = 0.36), consistent with the clinical hallmark of anxiety and depression comorbidity. In summary, although these variables exhibit a degree of clinical clustering, they retain sufficient statistical independence for collective inclusion in the development of the predictive model.

### 3.4 Selection of predictive features based on LASSO regression

**Figure 3:**
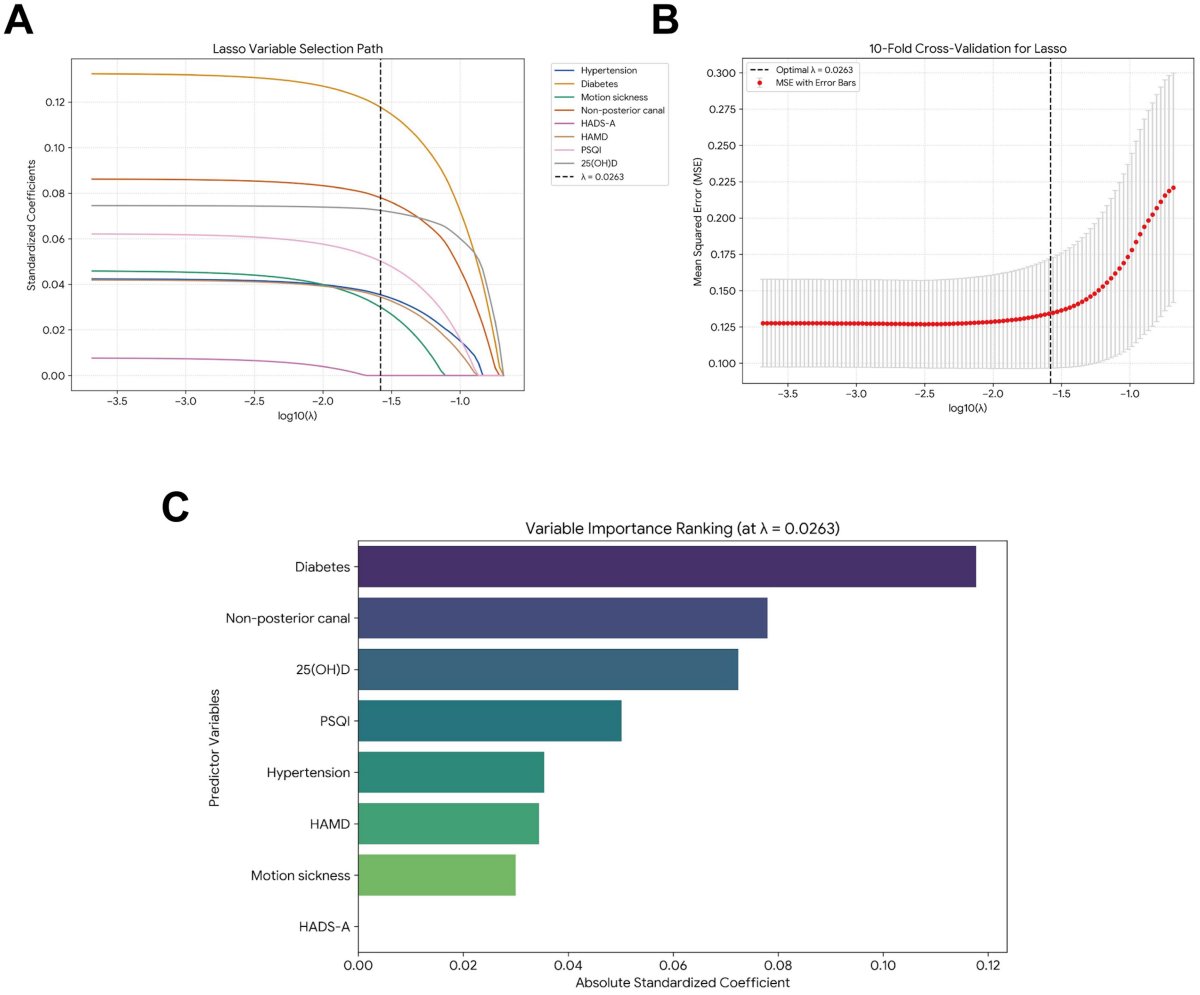
Feature selection and importance assessment based on the LASSO regression model **(A)** 10-fold cross-validation plot. The vertical axis represents the mean squared error (MSE), and the horizontal axis represents the logarithmic transformation of *λ* (log *λ*). **(B)** LASSO regression coefficient path plot. This plot illustrates the shrinkage trajectories of the standardized regression coefficients for each variable as the *λ* value changes. As *λ* decreases (moving from right to left), the model penalty weakens, and the coefficients of the characteristic variables are gradually released and stabilize. **(C)** Importance ranking of key risk factors. Variables are ranked in descending order based on the absolute values of their standardized coefficients at the optimal *λ*, reflecting the relative contribution of each variable to the outcome prediction (the coefficient for HADS-A has been compressed to zero).

To enhance the model’s generalizability, eight variables identified as statistically significant in the univariate analysis were incorporated into a LASSO regression model. The optimal penalty coefficient (*λ*) was determined via 10-fold cross-validation. As illustrated in Figure 3A, at the optimal *λ* value of 0.0263, the model achieved an ideal balance between predictive performance and sparsity. The coefficient profile plot displays the convergence trajectories of the regression coefficients for each variable as a function of the logarithm of *λ*(log *λ*) (Figure 3B). Under the constraint of the optimal *λ*, the coefficient for HADS-A was reduced to zero, indicating that its independent predictive contribution to BPPV recurrence was negligible after adjusting for other covariates; consequently, it was excluded from the final selection. Integrating these results with the correlation heatmap, which demonstrated no substantial multicollinearity among the variables, seven core risk factors were ultimately selected for inclusion in the subsequent multivariate logistic regression analysis. Furthermore, variable importance analysis revealed that diabetes mellitus, non-posterior semicircular canal involvement, and 25(OH)D deficiency represented the top three weighted predictors (Figure 3C).

### 3.5 Identification of independent risk factors for BPPV recurrence

**Figure 4:**
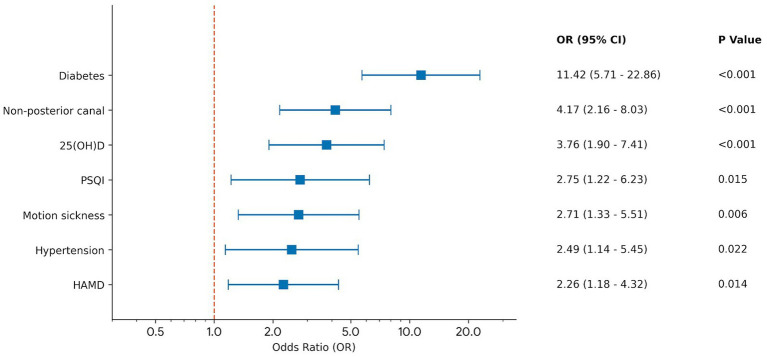
Forest plot of multivariate logistic regression analysis for risk factors associated with BPPV recurrence. The blue squares represent the odds ratio (OR), and the horizontal lines represent the 95% confidence interval (95% CI). The red vertical dashed line indicates OR = 1.0 (the null line), variables positioned to the right of this line indicate an increased risk of recurrence. All seven variables shown are independent risk factors for recurrence.

To further identify independent predictors of BPPV recurrence, the seven candidate variables identified through LASSO regression were incorporated into a multivariate logistic regression model for adjusted analysis. The results indicated that all seven variables served as independent risk factors for BPPV recurrence (all *p* < 0.05). Notably, diabetes mellitus exhibited the most robust association with recurrence risk, yielding an adjusted odds ratio (OR) of 11.42. This was followed by the anatomical type of the affected semicircular canal; specifically, patients with non-posterior semicircular canal involvement demonstrated a significantly elevated risk of recurrence compared to those with posterior canal involvement. Furthermore, serum 25(OH)D deficiency, impaired sleep quality (PSQI), a history of motion sickness, hypertension, and depressive symptoms (HAMD) were each significantly and positively associated with an increased risk of recurrence (all *p* < 0.05) (Figure 4).

### 3.6 Construction and performance evaluation of the BPPV recurrence prediction nomogram

**Figure 5:**
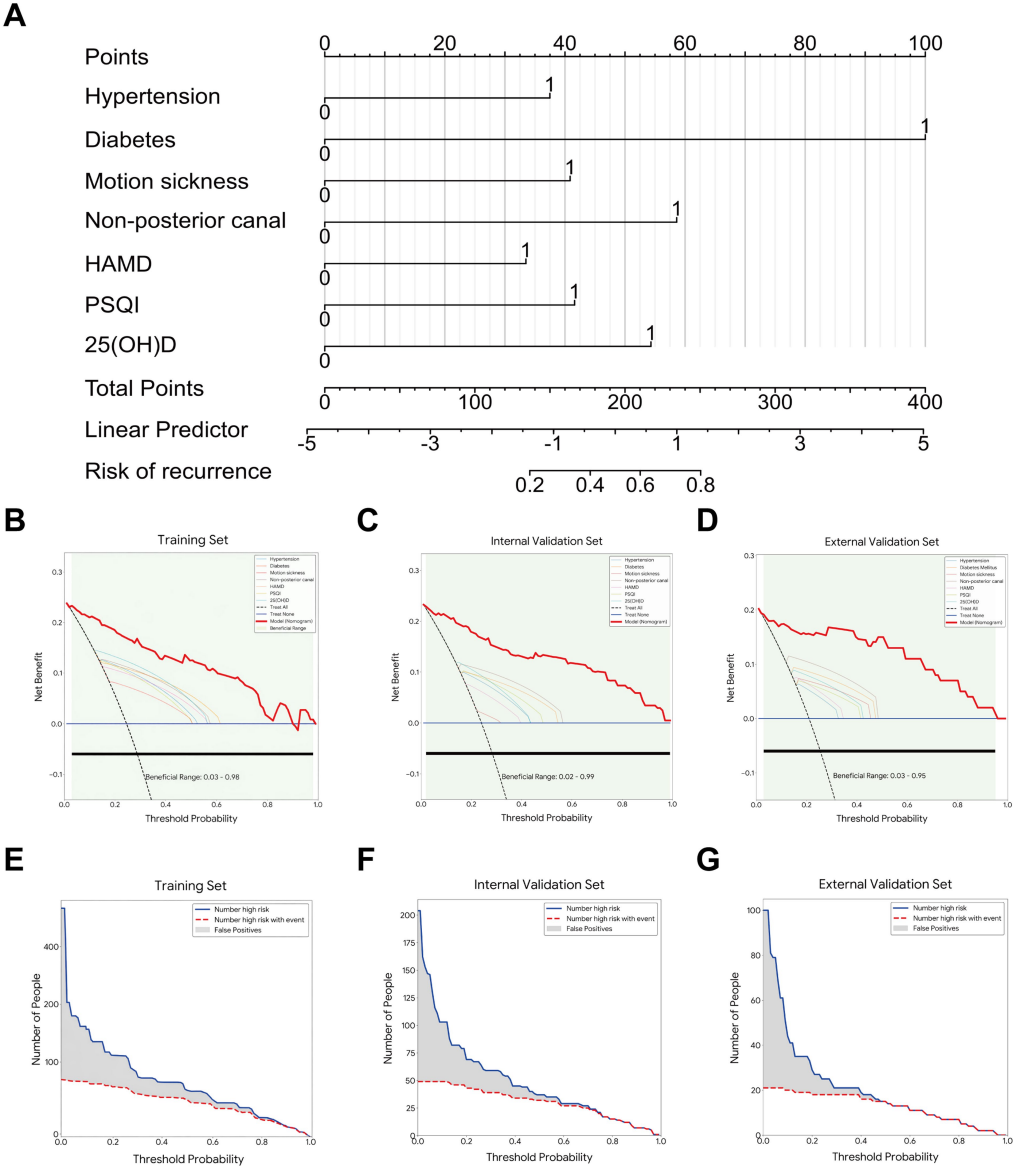
Nomogram for predicting the individualized risk of BPPV recurrence. **(A)** Nomogram for predicting the 1-year recurrence risk in patients with BPPV. DCA for the training set **(B)**, internal validation set **(C)**, and external validation set **(D)**. CIC for the training set **(E)**, internal validation set **(F)**, and external validation set **(G)**.

To facilitate a quantitative and individualized assessment of recurrence risk in patients with BPPV, a predictive nomogram was developed based on the seven independent risk factors identified through multivariate logistic regression analysis (Figure 5A). Within this model, each predictor was assigned a standardized score (ranging from 0 to 100 points) proportional to its regression coefficient. Notably, diabetes mellitus exerted the most substantial influence on recurrence risk and was consequently assigned the maximum score of 100 points. The remaining predictors were weighted as follows: non-posterior semicircular canal involvement (59 points), 25(OH)D deficiency (54 points), impaired sleep quality (PSQI; 42 points), a history of motion sickness (41 points), hypertension (37 points), and depressive symptoms (HAMD; 33 points).

The clinical utility of the model was further evaluated using DCA. Within the training set, the net benefit of utilizing this nomogram for clinical decision-making surpassed both the “treat-all” and “treat-none” strategies across a threshold probability range of 0.03–0.98 (Figure 5B). This clinical advantage was consistently validated in the internal validation set and the external validation set (Figures 5C,D). Furthermore, CIC demonstrated that across all three cohorts, the number of individuals identified as high-risk by the model closely aligned with the actual incidence of recurrence (Figures 5E–G). These findings support the potential clinical value and practical applicability of the proposed model.

### 3.7 Development and selection of the parsimonious multivariate predictive model

**Figure 6:**
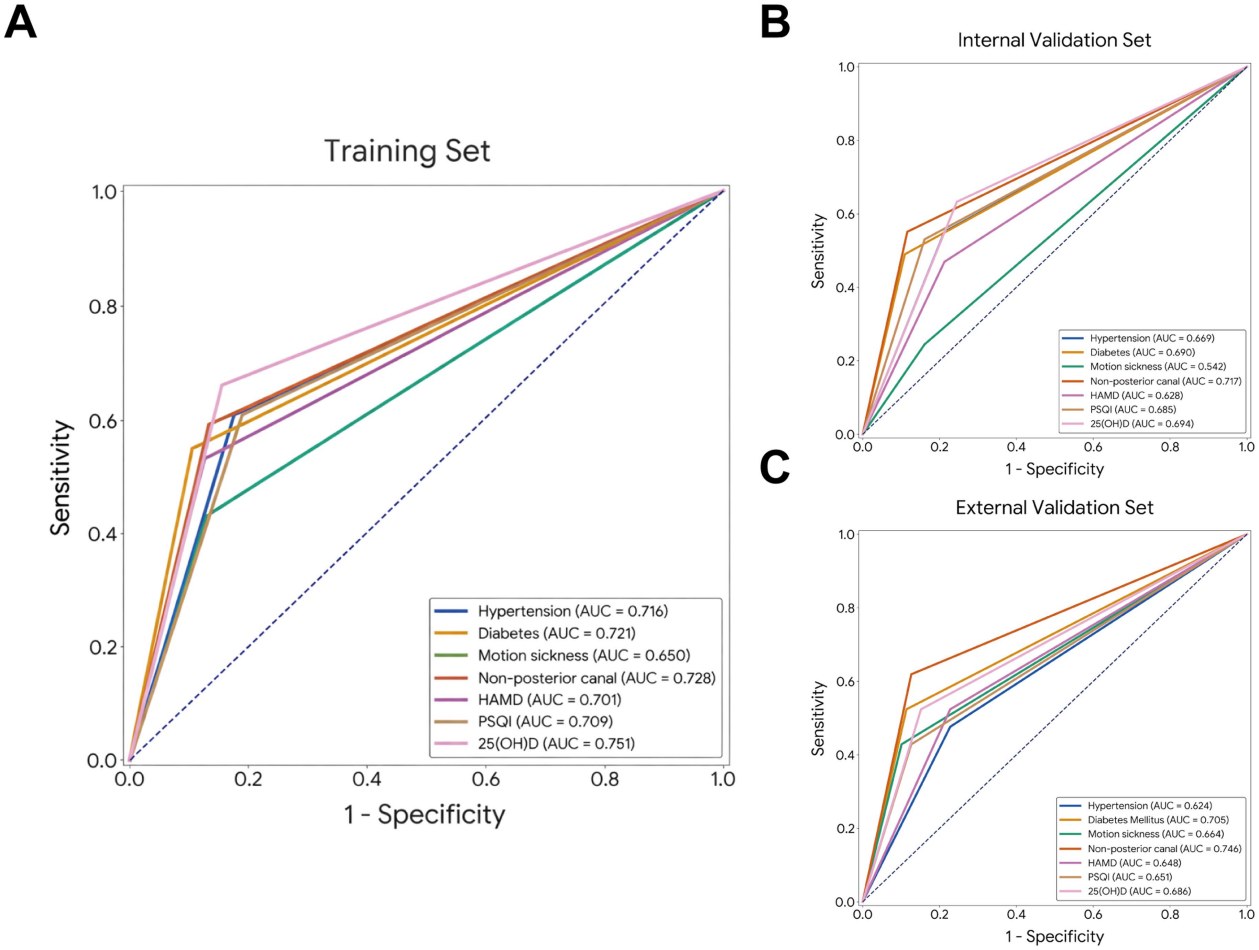
Univariate predictive performance of the seven independent risk factors across cohorts: **(A)** Training set; **(B)** internal validation set; **(C)** external validation set.

To balance predictive accuracy with clinical utility and reduce the data collection burden in primary care settings, we developed a “parsimonious model” as a streamlined alternative to the full-variable predictive model. Initially, the univariate predictive performance of the seven independent risk factors was evaluated across the training, internal validation, and external validation cohorts. Discriminatory power was quantified using the AUC (Figures 6A–C). Across all three datasets, although the specific hierarchical order of AUC values varied slightly, the top three predictors remained consistent: 25(OH)D deficiency, non-posterior semicircular canal involvement, and Diabetes Mellitus consistently demonstrated the most robust discriminatory power. As for the remaining four variables, although their hierarchical rankings exhibited marginal shifts across the internal and external validation cohorts relative to the training set, they consistently demonstrated moderate univariate predictive efficacy.

**Figure 7:**
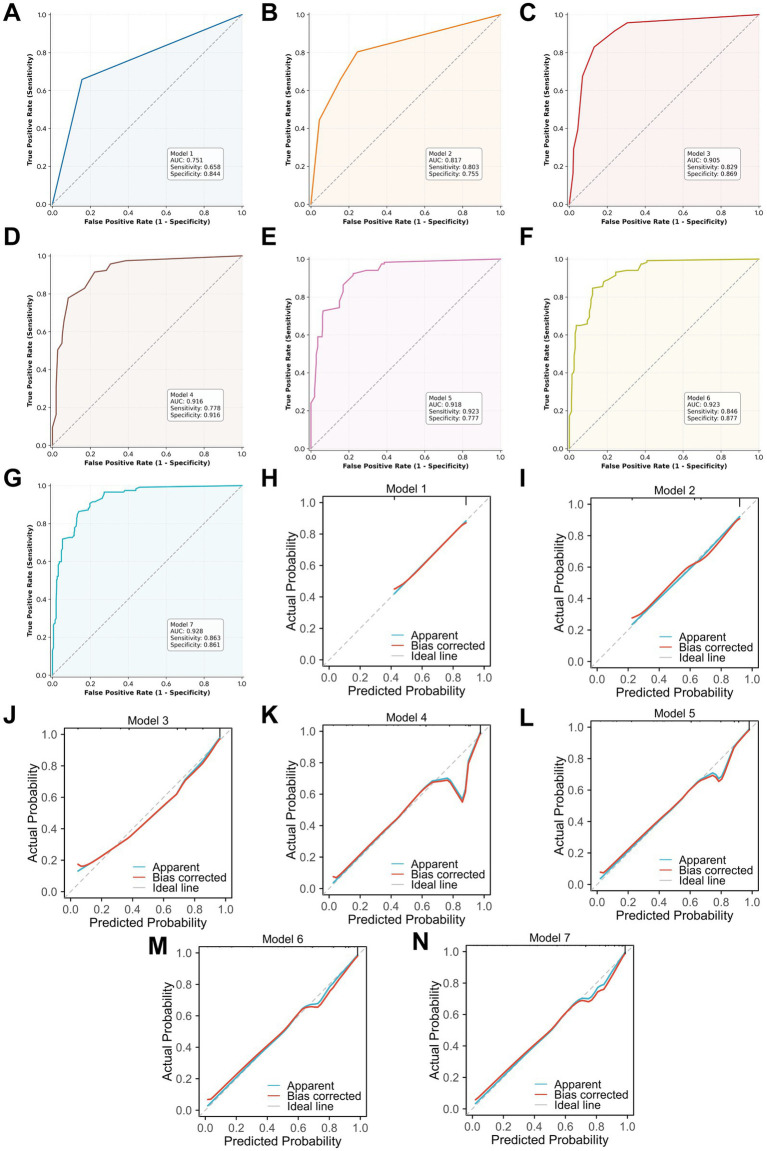
Performance evaluation and selection of predictive models for recurrence **(A–G)** ROC curves for Models 1 through 7. **(H–N)** Calibration curves for Models 1 through 7.

Based on variable importance, seven progressive logistic regression models were constructed by sequentially incorporating significant risk factors within the training set (Table 3). The results demonstrated a stepwise enhancement in model performance corresponding to the incremental inclusion of predictors (Figures 7A–G). Furthermore, the calibration curves for all seven models aligned closely with the ideal diagonal, indicating high concordance between predicted probabilities and observed recurrence outcomes (Figures 7H–N). Model 1 yielded an AUC of 0.751, however, the integration of non-posterior semicircular canal involvement and diabetes mellitus to form Model 3 resulted in a substantial performance leap, with the AUC increasing significantly from 0.817 (Model 2) to 0.905. Although the subsequent inclusion of additional variables (Models 4–7) yielded only marginal improvements in AUC, Model 3 was selected as the optimal predictive model, prioritizing parsimony and clinical efficiency. Comprising only three core variables, Model 3 achieved a sensitivity of 82.9% and a specificity of 86.9% in the training set. Compared to the full-variable model (Model 7), Model 3 retained over 97% of the predictive efficacy while substantially reducing the complexity of clinical data acquisition.

**Table 3:** Construction and variable composition of seven progressive logistic regression models for BPPV recurrence.

Model	Risk factors
Model 1	25(OH)D
Model 2	25(OH)D + non-posterior canal
Model 3	25(OH)D + non-posterior canal + diabetes
Model 4	25(OH)D + non-posterior canal + diabetes + hypertension
Model 5	25(OH)D + non-posterior canal + diabetes + hypertension + PSQI
Model 6	25(OH)D + non-posterior canal + diabetes + hypertension + PSQI + HAMD
Model 7	25(OH)D + non-posterior canal + diabetes + hypertension + PSQI + HAMD + motion sickness

To ensure full reproducibility, we report the complete statistical details of the final parsimonious model in Table 4, including beta coefficients, standard errors, adjusted odds ratios, 95% confidence intervals, and *p* values. All predictors were coded as binary variables (0 = absent/normal, 1 = present/abnormal). The logistic regression equation derived from the training set is: Logit(P) = −2.186 + 2.436 × Diabetes mellitus + 1.429 × Non-posterior canal involvement + 1.325 × 25(OH)D deficiency.

**Table 4:** Regression parameters of the final parsimonious model.

Predictor	*β* coefficient	Standard error	Adjusted OR	95% CI	*p*-value
Intercept	−2.186	0.245	—	—	—
Diabetes mellitus	2.436	0.312	11.42	6.28–20.74	<0.001
Non-posterior canal involvement	1.429	0.287	4.17	2.39–7.28	<0.001
25(OH)D deficiency	1.325	0.279	3.76	2.19–6.46	<0.001

*β: Logistic regression coefficient; OR, odds ratio; CI, confidence interval.

### 3.8 Validation of the parsimonious model in internal and external cohorts

**Figure 8:**
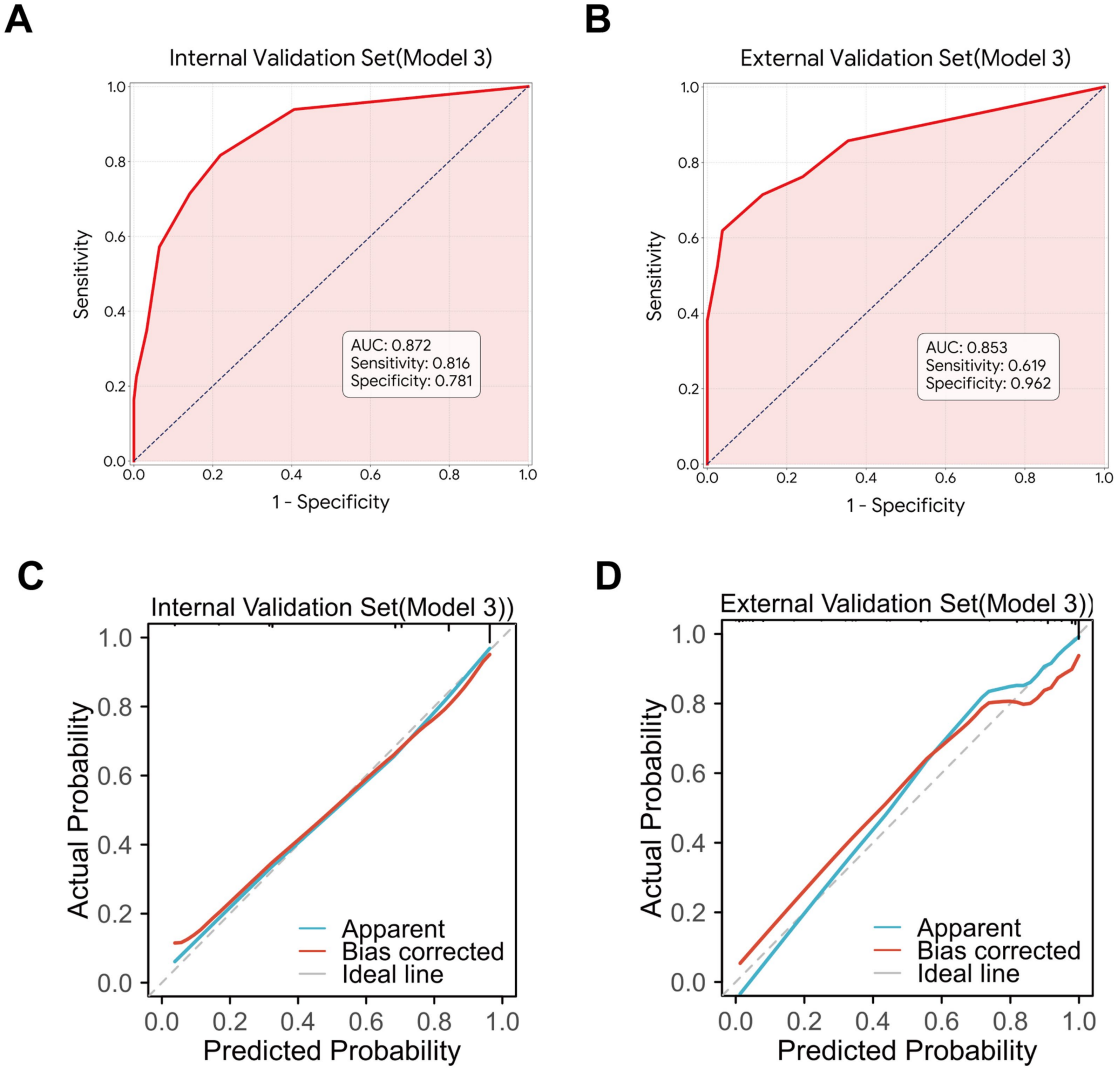
Evaluation of the discriminatory power and calibration of the parsimonious model (Model 3) in the internal and external validation cohorts. ROC curves for the internal validation set **(A)** and the external validation set **(B)**, respectively. Calibration curves for the internal validation set **(C)** and the external validation set **(D)**, respectively.

To evaluate the stability and generalizability of the parsimonious model comprising three core variables (Model 3), rigorous validation was conducted across both internal and external cohorts. Model 3 exhibited good discriminatory power in the internal validation set and maintained consistent performance in the external validation set. This consistency across independent populations supports the generalizability of the streamlined model. Notably, the parsimonious model excelled in identifying low-risk patients, maintaining a high specificity of approximately 78%–96% across both validation cohorts. This suggests that the model can reliably exclude patients at low risk for recurrence (Figures 8A,B).

Calibration was further assessed quantitatively using calibration intercept, calibration slope, and Brier score with 95% confidence intervals derived from 1,000 bootstrap resamples. In all cohorts, the calibration intercept was close to 0, the calibration slope was close to 1, and the Brier score was low, indicating good prediction accuracy. These quantitative results were consistent with the visual calibration curves, which demonstrated a high degree of alignment with the ideal diagonal for both the internal and external validation sets (Figures 8C,D). Furthermore, the Hosmer–Lemeshow goodness-of-fit test yielded non-significant results for both the internal validation set (*χ*^2^ = 0.575, *p* = 1.000) and the external validation set (*χ*^2^ = 4.561, *p* = 0.803). The findings that all *p*-values exceeded the 0.05 threshold indicate good concordance between the predicted probabilities and the observed clinical outcomes.

### 3.9 Clinical utility of the parsimonious model

**Figure 9:**
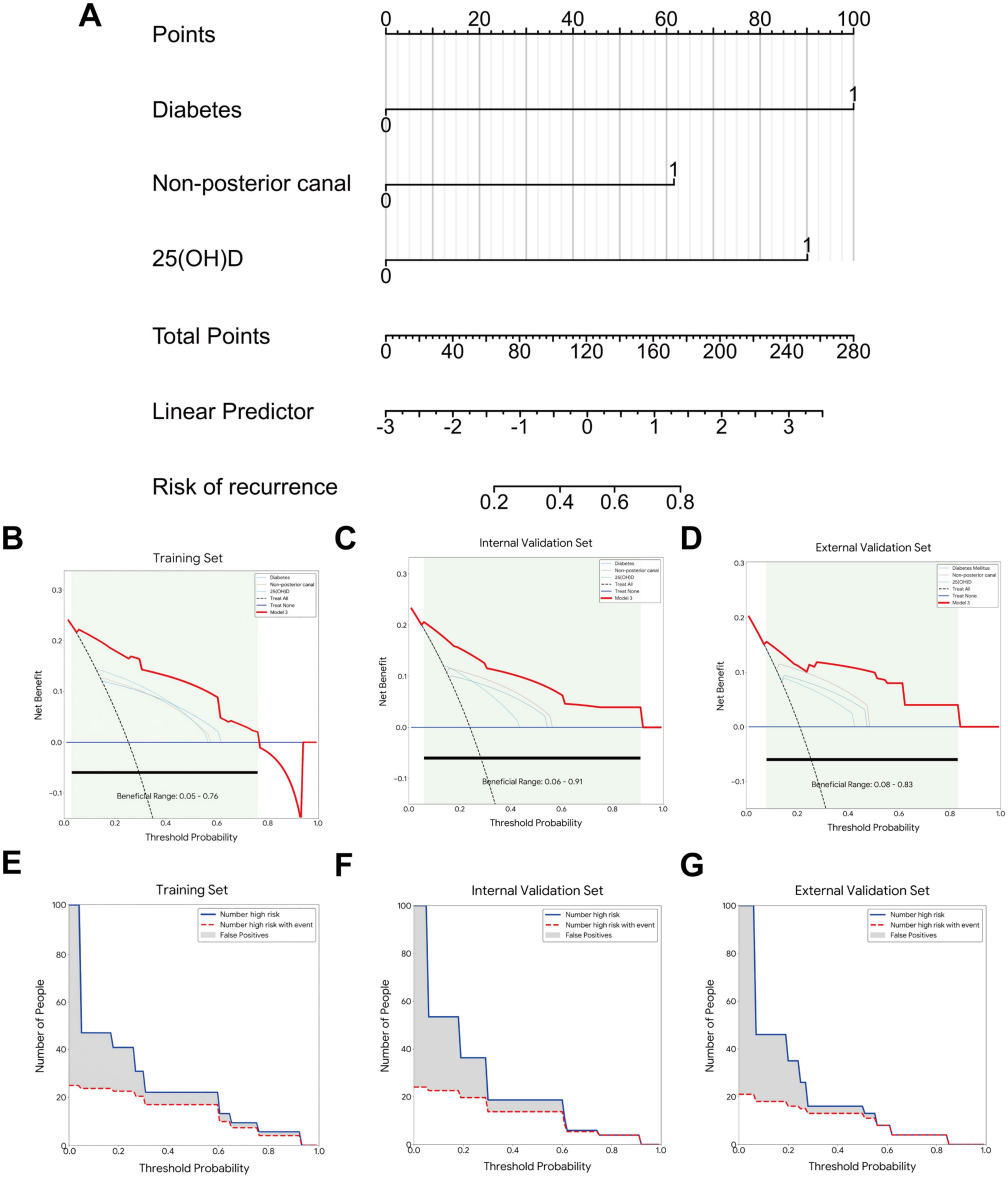
Clinical utility and predictive reliability of the parsimonious nomogram. **(A)** Nomogram for predicting the individualized risk of BPPV recurrence, established based on the optimal parsimonious model (Model 3). DCA evaluating the clinical net benefit of the nomogram in the training set **(B)**, internal validation set **(C)**, and external validation set **(D)**. CIC for the training set **(E)**, internal validation set **(F)**, and external validation set **(G)**.

To facilitate clinical implementation, a visualized nomogram for predicting the individualized risk of BPPV recurrence was developed based on the optimal parsimonious model (Model 3) (Figure 9A). Subsequently, Decision Curve Analysis (DCA) was performed to quantitatively evaluate the clinical utility of this nomogram. The results demonstrated that within the training set, the net benefit of implementing intervention strategies guided by the nomogram consistently surpassed those of the “treat-all” and “treat-none” strategies across a threshold probability range of 0.05 to 0.76 (Figure 9B). This clinical advantage was consistently observed across the validation cohorts, the benefit threshold range extended from 0.06 to 0.91 in the internal validation set (Figure 9C) and spanned 0.08 to 0.83 in the external validation set (Figure 9D). Notably, these threshold ranges encompass the observed overall recurrence rate of 23.97%, suggesting clinical relevance across a realistic spectrum of decision-making. Furthermore, CIC demonstrated the reliability of the model (Figures 9E–G). Across the training, internal validation, and external validation cohorts, the predicted high-risk population curves demonstrated high concordance with the observed recurrence curves under the primary risk thresholds.

## 4 Discussion

Based on clinical data from 780 patients with BPPV, this study systematically investigated the risk factors for post-repositioning recurrence and developed a parsimonious nomogram model suitable for potential clinical application. Our findings revealed an overall 1-year recurrence rate of 23.97%, which is consistent with recent epidemiological data (13.7%–48%) (26), supporting the representativeness of our study sample. Through LASSO regression dimensionality reduction and multivariate logistic regression analysis, seven independent risk factors were identified, leading to the derivation of a parsimonious model (Model 3) comprising diabetes mellitus, non-posterior semicircular canal involvement, and 25(OH)D deficiency. This streamlined model maintains good predictive performance and cross-center validation stability while substantially reducing the burden of clinical data collection. These results suggest that BPPV recurrence risk assessment does not necessitate exhaustive metrics; rather, precise stratification can be achieved by targeting the core “metabolic-anatomical-nutritional” axis.

The interaction between metabolic comorbidities and inner ear microcirculation plays a pivotal role in BPPV recurrence. Notably, diabetes mellitus was identified as the strongest predictor of recurrence in this study. The observed effect size is considerably greater than the approximately 3.2-fold risk previously reported by Wang et al. (24). This discrepancy may be attributed to differences in glycemic control status or disease duration within our cohort, and residual confounding may also contribute as we lacked detailed data on HbA1c levels, diabetic microvascular complications, and specific antidiabetic medication use. This finding aligns with the “vascular-otolith metabolism hypothesis.” Chronic hyperglycemia in diabetes leads to inner ear microangiopathy, causing ischemia, hypoxia, and local acidosis in the macular utriculi. These changes trigger the degeneration and detachment of otolith matrix proteins (27, 28). Additionally, hyperglycemia disrupts endolymphatic homeostasis by altering osmotic pressure, further undermining otolith stability after repositioning (29). Our findings suggest that for BPPV patients with comorbid diabetes, the objective of glycemic control should extend beyond the prevention of macrovascular complications to include the mitigation of vertigo recurrence. Future prospective studies incorporating these detailed metabolic parameters will help further clarify the causal relationship between diabetes and BPPV recurrence.

The association between 25(OH)D deficiency and BPPV recurrence has emerged as a significant research focus. We found that low serum 25(OH)D levels significantly elevated recurrence risk, demonstrating the highest predictive efficacy in univariate analysis. Otoliths are primarily composed of calcium carbonate crystals and glycoproteins, and their formation and maintenance are highly dependent on the regulation of calcium metabolism (30, 31). 25(OH)D receptors (VDRs) are widely distributed in inner ear epithelial cells, regulating the expression of calcium-binding proteins and endolymphatic calcium ion homeostasis (32, 33). 25(OH)D deficiency leads to reduced calcium absorption and compensatory elevations in parathyroid hormone, accelerating bone resorption while disrupting the otolith mineralization process. This compromises the structural integrity of the otoliths, rendering them fragile and susceptible to detachment (34). A randomized controlled trial by Jeong et al. (35) demonstrated that 25(OH)D and calcium supplementation could reduce BPPV recurrence by 45%. Our nomogram assigns substantial weight to 25(OH)D deficiency, highlighting the potential value of serum 25(OH)D screening and supplementation in the secondary prevention of BPPV.

Regarding anatomical factors, this study observed that patients with non-posterior semicircular canal (primarily horizontal canal) involvement exhibited a significantly higher recurrence risk than those with posterior canal involvement. This finding may be attributed to anatomical structure and the therapeutic challenges associated with repositioning. Previous research has largely focused on posterior canal BPPV, often overlooking prognostic disparities in non-posterior subtypes. Although repositioning for horizontal canal BPPV (HC-BPPV) is effective, frequent anatomical variations and the propensity for “canal conversion” hinder the complete clearance of residual debris, creating a latent source for recurrence (36). Furthermore, Imai et al. suggested that non-posterior canal involvement often indicates more extensive vestibular degeneration (37). By incorporating this anatomical feature into its core architecture, our parsimonious model addresses a lacuna in previous models regarding subtype differentiation, suggesting that non-posterior BPPV patients warrant more intensive follow-up.

Notably, although psychological and sleep indicators were excluded from the final parsimonious model to prioritize clinical utility, full-variable analysis confirmed that HAMD and PSQI scores were independent risk factors for recurrence. This underscores the role of the “ear-brain axis” interaction in the progression of BPPV (38–40). Chronic depression and sleep deprivation can lead to autonomic dysfunction and vasoconstriction, exacerbating inner ear ischemia (41, 42). Conversely, vestibular dysfunction itself can trigger secondary psychiatric symptoms, heightening a patient’s subjective perception of vertigo and increasing the likelihood of reported recurrence (43, 44). Therefore, for patients with significant emotional distress, canalith repositioning alone may be insufficient; multidisciplinary strategies involving psychological intervention and sleep management are essential.

Compared to previous complex models incorporating numerous variables (e.g., age, gender, hyperlipidemia), the parsimonious model developed in this study offers notable advantages for potential clinical implementation. Its minimal variable requirements significantly enhance feasibility in primary care settings. Furthermore, the broad DCA threshold ranges observed across all three cohorts suggest that the model could, in principle, accommodate a wide spectrum of clinical risk tolerance. However, the specific management strategies corresponding to different threshold ranges remain to be prospectively validated. For example, one could hypothesize that at low thresholds (5%–15%), the nomogram might inform broad, low-cost measures such as universal 25(OH)D screening and patient education; at intermediate thresholds (15%–30%), it could potentially support risk-stratified triage (e.g., intensified follow-up for high-risk patients while sparing low-risk patients from unnecessary intervention); and at high thresholds (50%–76%), it might help identify candidates for resource-intensive strategies such as pharmacological vitamin D supplementation with serial monitoring or multidisciplinary co-management. These proposed pathways should be viewed as hypotheses generated by the model rather than as established clinical protocols. Prospective intervention studies are needed to determine whether model-guided management improves patient outcomes compared to standard care.

Despite the advantages of multicenter validation, this study has several limitations. First, its retrospective nature may introduce selection and information bias. Second, the 1-year follow-up period does not account for long-term recurrence risk. Third, the external validation cohort, while independent and geographically distinct, was relatively small (*n* = 100) with only 21 recurrence events, which limits the precision of performance estimates, particularly for calibration metrics and decision curve analysis. Although the external AUC remained 0.853, the modest number of events precluded precise estimation of sensitivity and calibration parameters. Additionally, objective vestibular parameters such as videonystagmography (VNG) were not incorporated into the model. Fourth, the strict exclusion criteria—while necessary to reduce confounding—limit the model’s applicability to complex dizziness populations. Patients with coexisting vestibular migraine, Ménière’s disease, persistent postural-perceptual dizziness (PPPD), anxiety disorders, age-related imbalance, and cerebrovascular or cervical comorbidities were excluded. In clinical reality, such conditions frequently coexist with BPPV and may independently contribute to positional symptom recurrence. Consequently, the model’s predictive performance in unselected, multimorbid dizziness populations remains to be established. Future research should involve larger multicenter prospective cohorts that include patients with common otological and neuropsychiatric comorbidities. This would allow evaluation of the model’s real-world generalizability. Objective indicators—such as vestibular evoked myogenic potentials (VEMPs)—should also be incorporated to further refine predictive performance.

## 5 Conclusion

Diabetes mellitus, non-posterior semicircular canal involvement, and 25(OH)D deficiency were identified as core risk factors for BPPV recurrence in this cohort. The nomogram developed in this study is streamlined and intuitive, providing a preliminary tool for individualized risk stratification. Its clinical utility in guiding preventive strategies awaits confirmation in prospective studies.

## Data Availability

The original contributions presented in the study are included in the article/supplementary material, further inquiries can be directed to the corresponding author.

## References

[1] von Brevern M Bertholon P Brandt T Fife T Imai T Nuti D . Benign paroxysmal positional vertigo: diagnostic criteria. J Vestib Res. (2015) 25:105–17. doi: 10.3233/ves-150553 26756126

[2] Neuhauser HK. The epidemiology of dizziness and vertigo. Handb Clin Neurol. (2016) 137:67–82. doi: 10.1016/b978-0-444-63437-5.00005-4, 27638063

[3] Bhattacharyya N Gubbels SP Schwartz SR Edlow JA El-Kashlan H Fife T . Clinical practice guideline: benign paroxysmal positional vertigo (update). Otolaryngol Head Neck Surg. (2017) 156:S1–S47. doi: 10.1177/0194599816689667, 28248609

[4] Parnes LS Agrawal SK Atlas J. Diagnosis and management of benign paroxysmal positional vertigo (BPPV). CMAJ. (2003) 169:681–93. 14517129 PMC202288

[5] Hilton MP Pinder DK. The epley (canalith repositioning) manoeuvre for benign paroxysmal positional vertigo. Cochrane Database Syst Rev. (2014) 2014:Cd003162. doi: 10.1002/14651858.CD003162.pub3, 11869655

[6] Power L Murray K Szmulewicz DJ. Characteristics of assessment and treatment in benign paroxysmal positional vertigo (BPPV). J Vestib Res. (2020) 30:55–62. doi: 10.3233/ves-190687, 31839619 PMC9249279

[7] Kong TH Song MH Shim DB. Recurrence rate and risk factors of recurrence in benign paroxysmal positional vertigo: a single-center long-term prospective study with a large cohort. Ear Hear. (2022) 43:234–41. doi: 10.1097/aud.0000000000001093, 34320525

[8] Prokopakis E Vlastos IM Tsagournisakis M Christodoulou P Kawauchi H Velegrakis G. Canalith repositioning procedures among 965 patients with benign paroxysmal positional vertigo. Audiol Neurootol. (2013) 18:83–8. doi: 10.1159/000343579, 23147839

[9] Cengiz DU Demir İ Demirel S Can Çolak S Emekçi T Bayındır T. Investigation of the relationship between BPPV with anxiety, sleep quality and falls. Turk Arch Otorhinolaryngol. (2022) 60:199–205. doi: 10.4274/tao.2022.2022-8-6, 37456598 PMC10339271

[10] Yeo SC Ahn SK Lee HJ Cho HJ Kim SW Woo SH . Idiopathic benign paroxysmal positional vertigo in the elderly: a long-term follow-up study. Aging Clin Exp Res. (2018) 30:153–9. doi: 10.1007/s40520-017-0763-2, 28429295

[11] Zhou R Wang H Shan J Li C Han L. Clinical features of benign paroxysmal positional vertigo in the elderly. Front Neurol. (2025) 16:1623914. doi: 10.3389/fneur.2025.1623914, 40589989 PMC12206830

[12] Bayrak AF Çetin B Doğan M. Investigation of serum calcium and vitamin D levels in patients with refractory benign paroxysmal positional vertigo. Turk Arch Otorhinolaryngo. (2025) 63:179–84. doi: 10.4274/tao.2025.2025-7-1, 41445113 PMC12746168

[13] Liu J Liu J Dai Y He F Zhai H. Research progress on risk factors of recurrence of benign paroxysmal positional vertigo. Front Neurol. (2025) 16:1735898. doi: 10.3389/fneur.2025.1735898, 41603001 PMC12832348

[14] Micarelli A Granito I Micarelli RX Alessandrini M. The impact of hypertension and related risk factors on the onset and resolution rates of benign paroxysmal positional vertigo recurrence: a 6-year retrospective study. Neurol Int. (2025) 17:82. doi: 10.3390/neurolint17060082, 40559321 PMC12196472

[15] Bener A Erdoğan A Üstündağ ÜV. The impact of serums calcium 25-hydroxy vitamin D, ferritin, uric acid, and sleeping disorders on benign paroxysmal positional vertigo patients. Audiol Res. (2024) 14:640–8. doi: 10.3390/audiolres14040054, 39051198 PMC11270364

[16] Saeed Al-Rawi TS Al-Ani RM. Vitamin D deficiency and the risk of recurrent benign paroxysmal positional vertigo. Cureus. (2024) 16:e52433. doi: 10.7759/cureus.52433, 38371108 PMC10870803

[17] Tang B Luo M Wang D He Y Zhang C Yu X. The synergistic interaction of vitamin D deficiency and insomnia on dizziness-related handicap in patients with benign paroxysmal positional vertigo. Front Neurol. (2025) 16:1656528. doi: 10.3389/fneur.2025.1656528, 41170330 PMC12568406

[18] Chandrakala S Doreswamy SM. Management protocol for the unilateral posterior canal - benign paroxysmal positional vertigo - a prospective observational study. Indian J Otolaryngol Head Neck Surg. (2024) 76:5464–9. doi: 10.1007/s12070-024-05006-x, 39558983 PMC11569298

[19] Lee SJ Hur Y. Living with the fear of recurrence in benign paroxysmal positional vertigo. Health Expect. (2026) 29:e70551. doi: 10.1111/hex.70551, 41489048 PMC12766396

[20] Ge X Yang G Wu H Liu L Zhou M Zhu A . Prognostic factors and nomogram-based prediction models for colorectal Cancer patients with synchronous peritoneal metastasis undergoing cytoreductive surgery: a retrospective cohort study. Cancer Med. (2026) 15:e71464. doi: 10.1002/cam4.71464, 41452583 PMC12742547

[21] Yan Q Zhang Z Li R Xue J Yan X Song J. Clinical and nutritional-inflammatory biomarkers-based nomogram predicts survival in recurrent or metastatic cervical cancer treated with immune checkpoint inhibitors. Ann Med. (2026) 58:2603780. doi: 10.1080/07853890.2025.2603780, 41466422 PMC12777797

[22] Zhang B Yang Q Sun H Liu M Liang H Ji H. Development and validation of a nomogram for the overall survival of patients with locally advanced prostate cancer treated with radiotherapy and surgery. Aging Male. (2026) 29:2609400. doi: 10.1080/13685538.2025.2609400, 41503827

[23] Pan Q Li B Zou K Zhang J Wang Y Tang X. Risk factors and a nomogram model for recurrence of benign paroxysmal positional vertigo: a multicenter cross-sectional study. Front Neurol. (2025) 16:1542090. doi: 10.3389/fneur.2025.1542090, 40248016 PMC12003364

[24] Wang P. Risk factors for recurrence in benign paroxysmal positional vertigo patients and construction of a predictive nomogram. Medicine (Baltimore). (2025) 104:e44498. doi: 10.1097/md.0000000000044498, 40958245 PMC12440414

[25] Surgery CSoOHaN. Guideline of diagnosis and treatment of benign paroxysmal positional vertigo (2017). Chin J Otorhinolaryngol Head Neck Surg. (2017) 52:173–7. doi: 10.3760/cma.j.issn.1673-0860.2017.03.003 28395487

[26] Sfakianaki I Binos P Karkos P Dimas GG Psillas G. Risk factors for recurrence of benign paroxysmal positional vertigo. A clinical review. J Clin Med. (2021) 10:10. doi: 10.3390/jcm10194372, 34640391 PMC8509726

[27] Gioacchini FM Albera R Re M Scarpa A Cassandro C Cassandro E. Hyperglycemia and diabetes mellitus are related to vestibular organs dysfunction: Truth or suggestion? A literature review. Acta Diabetol. (2018) 55:1201–7. doi: 10.1007/s00592-018-1183-2, 29936650

[28] Kumar P Singh NK Apeksha K Ghosh V Kumar RR Kumar MB. Auditory and vestibular functioning in individuals with type-2 diabetes mellitus: a systematic review. Int Arch Otorhinolaryngol. (2022) 26:e281–8. doi: 10.1055/s-0041-1726041, 35602282 PMC9122769

[29] Pålbrink AK In’t Zandt R Magnusson M Degerman E. Betahistine prevents development of endolymphatic hydrops in a mouse model of insulin resistance and diabetes. Acta Otolaryngol. (2023) 143:127–33. doi: 10.1080/00016489.2023.2171116, 36735299

[30] Huang S Qian S. Advances in otolith-related protein research. Front Neurosci. (2022) 16:956200. doi: 10.3389/fnins.2022.956200, 35958995 PMC9361852

[31] Lundberg YW Xu Y Thiessen KD Kramer KL. Mechanisms of otoconia and otolith development. Dev Dyn. (2015) 244:239–53. doi: 10.1002/dvdy.24195, 25255879 PMC4482761

[32] Yamauchi D Nakaya K Raveendran NN Harbidge DG Singh R Wangemann P . Expression of epithelial calcium transport system in rat cochlea and vestibular labyrinth. BMC Physiol. (2010) 10:1. doi: 10.1186/1472-6793-10-1, 20113508 PMC2825184

[33] Zhang S Xing J Gong Y Li P Wang B Xu L. Downregulation of VDR in benign paroxysmal positional vertigo patients inhibits otolith-associated protein expression levels. Mol Med Rep. (2021) 24:10. doi: 10.3892/mmr.2021.12230 34165161

[34] Miśkiewicz-Orczyk K Pluskiewicz W Kos-Kudła B Misiołek M. Assessment of osteoporosis and vitamin D3 deficiency in patients with idiopathic benign paroxysmal positional Vertigo (BPPV). Medicina (Kaunas). (2023) 59:862. doi: 10.3390/medicina59050862, 37241094 PMC10221187

[35] Jeong SH Kim JS Kim HJ Choi JY Koo JW Choi KD . Prevention of benign paroxysmal positional vertigo with vitamin D supplementation: a randomized trial. Neurology. (2020) 95:e1117–25. doi: 10.1212/wnl.0000000000010343, 32759193

[36] Rajguru SM Ifediba MA Rabbitt RD. Biomechanics of horizontal canal benign paroxysmal positional vertigo. J Vestib Res. (2005) 15:203–14. doi: 10.3233/VES-2005-15404, 16286702 PMC2716391

[37] Imai T Takeda N Ikezono T Shigeno K Asai M Watanabe Y . Classification, diagnostic criteria and management of benign paroxysmal positional vertigo. Auris Nasus Larynx. (2017) 44:1–6. doi: 10.1016/j.anl.2016.03.013, 27174206

[38] Fu W Bai Y He F Wei D Wang Y Shi Y . The association between precuneus function and residual dizziness in patients with benign paroxysmal positional vertigo. Front Neurol. (2022) 13:828642. doi: 10.3389/fneur.2022.828642, 35493847 PMC9039311

[39] Széphelyi K Kóra S Orsi G Tollár J. Structural brain abnormalities, diagnostic approaches, and treatment strategies in vertigo: a case-control study. Neurol Int. (2025) 17:146. doi: 10.3390/neurolint17090146, 41002934 PMC12472988

[40] Zhu Q Chen W Cui Y Wu J Shu L Sun X . Structural and functional changes in the cerebellum and brainstem in patients with benign paroxysmal positional vertigo. Cerebellum. (2021) 20:804–9. doi: 10.1007/s12311-021-01237-8, 33547587

[41] Cherubini JM Cheng JL Williams JS MacDonald MJ. Sleep deprivation and endothelial function: reconciling seminal evidence with recent perspectives. Am J Physiol Heart Circ Physiol. (2021) 320:H29–35. doi: 10.1152/ajpheart.00607.2020, 33064569

[42] Liu LF Wang YW Sun JC Wang YK Tan X Wang WZ. Sleep deprivation reduces the baroreflex sensitivity through elevated angiotensin (Ang) II subtype 1 receptor expression in the nucleus tractus solitarii. Front Neurosci. (2024) 18:1401530. doi: 10.3389/fnins.2024.1401530, 38741786 PMC11089155

[43] Shu Y Liao N Fang F Shi Q Yan N Hu Y. The relationship between psychological conditions and recurrence of benign paroxysmal positional vertigo: a retrospective cohort study. BMC Neurol. (2023) 23:137. doi: 10.1186/s12883-023-03169-8, 37004007 PMC10064541

[44] Staab JP. Functional and psychiatric vestibular disorders. Handb Clin Neurol. (2016) 137:341–51. doi: 10.1016/b978-0-444-63437-5.00024-8, 27638082

